# Unveiling hotspots and trends in hip arthroscopy research: A bibliometric and visualized analysis (1900–2022)

**DOI:** 10.1097/MD.0000000000038198

**Published:** 2024-05-24

**Authors:** Jinlong Tian, Yanlei Li, Yu Tong, Lichen Ji, Wei Zhang, Xugang Zhong, Senbo Zhu, Yao Kang, Qing Bi

**Affiliations:** a Center for Rehabilitation Medicine, Department of Orthopedics, Zhejiang Provincial People’s Hospital (Affiliated People’s Hospital, Hangzhou Medical College), Hangzhou, China; b Bengbu Medical College, Bengbu, Anhui, China; c Department of Joint Surgery, Shanghai East Hospital, Tongji University School of Medicine, Shanghai, China; d Department of Orthopedics, Taizhou Hospital of Zhejiang Province affiliated to Wenzhou Medical University, Linhai, Zhejiang, China.

**Keywords:** bibliometric, femoroacetabular impingement, hip arthroscopy, VOSviewer

## Abstract

Over the past 10 years, hip arthroscopy has been increasingly employed to effectively diagnose and safely treat a range of hip pathologies. With research related to hip arthroscopy continually expanding, the number of articles connected with hip arthroscopy has also consistently grown. We aimed to investigate trends and hotspots in hip arthroscopy-related research, and analyze the top 100 most-cited articles on hip arthroscopy. We searched for (“hip arthroscopy”) AND (“article” OR “review”) AND “English” in the Web of Science database from 1900 to 2022, which was used to obtain all publications relating to hip arthroscopy. Distribution of country, affiliated institution, journal, authors, citation frequency and keywords were analyzed using VOSviewer. A total of 1094 articles were selected from the Web of Science Core Collection (WoSCC) from 1900 to 2022. The number of publications concerning hip arthroscopy displayed an ascending trend over time. Among the countries, the United States emerged as the largest contributor to the number of articles. The highest prolific institution was American Hip Institute. Among the journals, the highest-ranking journal was “*Arthroscopy-the Journal of Arthroscopic and Related Surgery*,” with 8316 citation counts and 262 articles. The area of greatest research interest was diagnosis and therapy in the field. The scientific articles on the subject of hip arthroscopy have risen continuously in recent years. The United States was the most influential country and made the most significant contributions to this field globally. We identified the research direction and trend for the first time and provided the most recent bibliometric analysis on hip arthroscopy, which may assist researchers in conducting studies on hip arthroscopy.

## 1. Introduction

With the advancement of endoscopy and minimally invasive techniques, hip arthroscopy is gaining increasingly significant importance in the field of joint surgery. Hip arthroscopy, a minimally invasive technique, is utilized to examine the internal structure and lesions of the hip joint. This technology allows visualization of the hip joint structure such as acetabular labrum, femoral and acetabular cartilage surface, fovea, ligaments, synovium and extraarticular trochanter.^[1]^ According to related research reports, during the period of 2011 to 2016, hip surgeries increased 2.6 times, and the application of hip arthroscopy increased by 310%.^[2]^ Furthermore, hip arthroscopy can significantly reduce morbidity and the risk of neurovascular injury and also shorten the recovery period compared to conventional hip surgery.^[3,4]^ The benefits of hip arthroscopy are substantial, with a 2-year follow-up study showing that it can significantly improve symptoms in treating femoral acetabular impingement syndrome (FAIS).^[5]^ With the increment in the number of hip arthroscopic surgery and its indications,^[6,7]^ its related researches are augmenting day by day. Despite these studies, the overall trends and research focus on hip arthroscopy remain unclear. Thus, we use bibliometrics method for comprehensive analysis.

Bibliometrics, suggested by American bibliographers in 1969, is applied to statistically analyze the different information of books and other communication media.^[8,9]^ Its strength lies in understanding the research dynamics in a specific field, and gives us a clearer picture of the distribution of authors, countries and journals in the study field, and may predict the trend of future research.^[10,11]^ Bibliometrics is widely used in the medical field, such as: myasthenia gravis, Biomechanics of lumbar intervertebral discs, brain-computer interface technology, robotic surgery, multiple myeloma bone diseases.^[12–16]^ Given the advantage of bibliometrics and the need to understand the current state of hip arthroscopy research, we conduct a bibliometric study related to hip arthroscopy.

Our aim is to analyze the articles related to hip arthroscopy, explore the trend of studies, and help researchers quickly understand the research highlights related to hip arthroscopy. Till date, there has been only one review linked to the 50 most-cited articles of hip arthroscopy. However, this publication did not include author, keywords, research trend, and country collaboration. We endeavor to evaluate the articles on hip arthroscopy from various aspects including developing trends and direction of hip arthroscopy, research highlight, overall keywords, collaborating institutions, and countries. We hope that this study provides some guidance for future researchers in hip arthroscopy.

## 2. Material and methods

On April 14, 2022, we collected the information for our study from the Web of Science Core Collection (WoSCC). The WoSCC, widely used for bibliometric analysis, consists of an extensive collection of journals, authoritative books, and representative articles. The research methodology was as follows: Title = hip arthroscopy AND Document type = (article OR review) AND Language = English AND Time span = 1900 to 2022.

### 2.1. Analytical tools

To analyze our study, we applied VOSviewer (version 1.6.18) (Leiden University, Netherlands), Scimago Graphica (version1.0.38), CiteSpace (version 6.2.R7), Pajek (version 5.18) and Microsoft Office Excel 2019 (Microsoft, USA). VOSviewer, a user-friendly software tool, runs on the Java platform and is used to visualize and construct bibliometric networks. These networks may comprise researchers, countries and research organizations, keywords or scientific journals, connected by co-authorship, Co-occurrence, Citation, Bibliographic coupling, or Co-citation. VOSviwer basically uses the default values for bibliometric analysis, and if there are any special changes, they are detailed in the figure legend.

We utilized the software called Scimago Graphica, designed for exploring, visually communicating, and making sense of data, as a novel approach. Pajek is software for creating and analyzing networks of various types. Scimago Graphica and Pajek were used to further analysis of the data derived from the VOSviewer. CiteSpace is also considered high-quality software in the bibliometric field, thus it is utilized for data analysis.

We employed Microsoft Office Excel 2019 to extract and analyze the relevant features of literature, such as title, author, journal, country, organization, publication years, total citations or journal impact factors, and create tables and figures.

### 2.2. Data extraction

After formulating the search strategy, 3 authors independently extracted the literature and bibliometric indicators from the data, and discussed any divergences until the consensus was reached. All of the data came from the WoSCC database in plain and Excel format and were extracted and analyzed using VOSviewer and Microsoft Office Excel 2019.

## 3. Results

### 3.1. Publication trend

**Figure 1.**
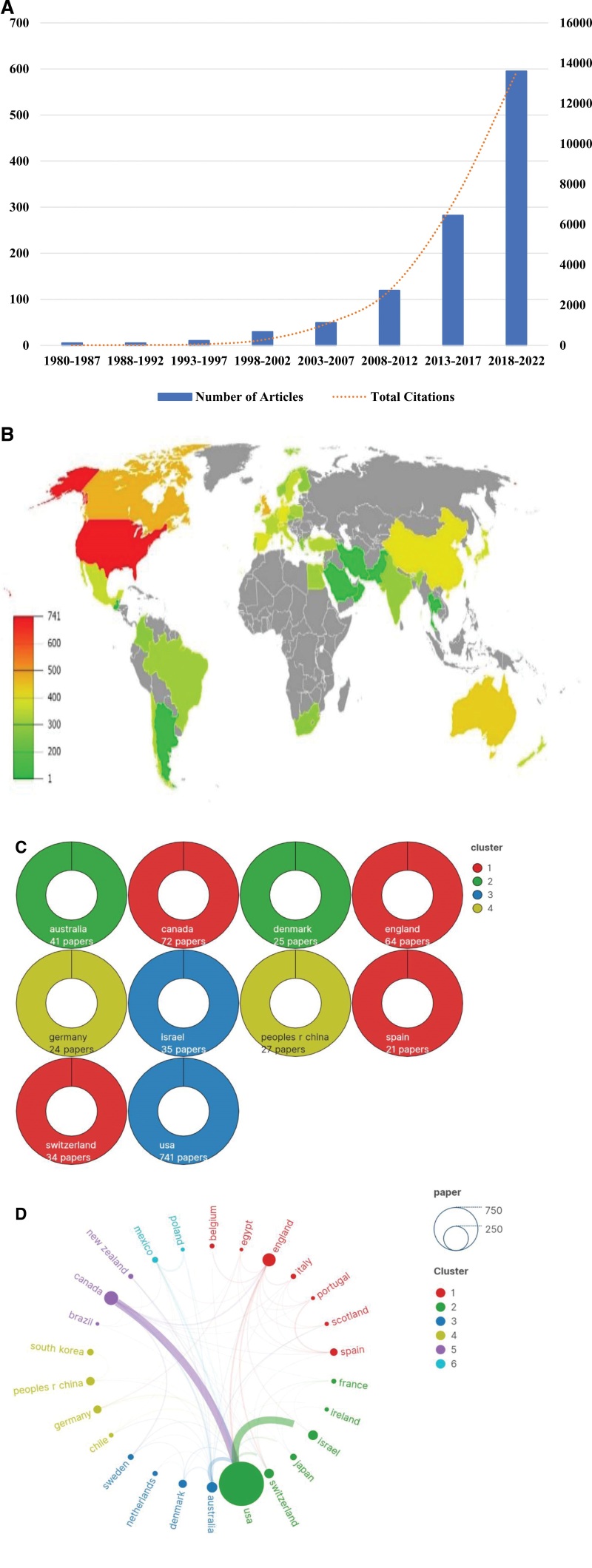
Overview of publications. (A) Trends in the number of publications and citations on hip arthroscopy research from 1900 to 2022. (B) Distribution of publications. (C) The top 10 most active countries, the same color represent the same grouping. (D) Network map of country co-authorship, Minimum number of documents of a country: 5, Of the 46 countries, 27 meet the threshold, The country of Turkey was excluded as it exhibited no connection with other countries.

A total of 1094 documents of hip arthroscopy research were identified in the WoSCC database. Generally, the number of publications concerning hip arthroscopy has increased between 1980 and 2022 (Fig. 1A). From 1980 to 1992, the quantity of publications was the lowest, with research on hip arthroscopy even coming to a standstill. The number of publications experienced a steady increase of 109 between 1993 and 2012, indicating that researchers started paying more attention to hip arthroscopy. From 2013 to 2022, publication outputs have seen a steep ascent.

### 3.2. Distribution of country

The articles hail from 46 countries with the USA (n = 741) and Canada (n = 72) contributing significantly to the overall number of articles, 3 times that of other countries (Fig. 1B and C). A visual network creating using VOSviewer and Scimago Graphica shows collaboration between countries (n = 26) based on the co-authorship. Only when those countries published 5 or more articles are they linked, and the size of the nodes represents the impact of the countries. the map is color-coded to represent different research directions. The proximity between nodes and the thickness of lines reflect the extent of cooperation between countries. The strongest linkage is observed between the USA and Israel (28), followed by between the USA and Canada (25.75), the USA and Australia (9.17), and the USA and Switzerland (7.25). Evidently, the USA has exerted an extensive influence on hip arthroscopy research (Fig. 1D).

### 3.3. Institution distribution

**Figure 2.**
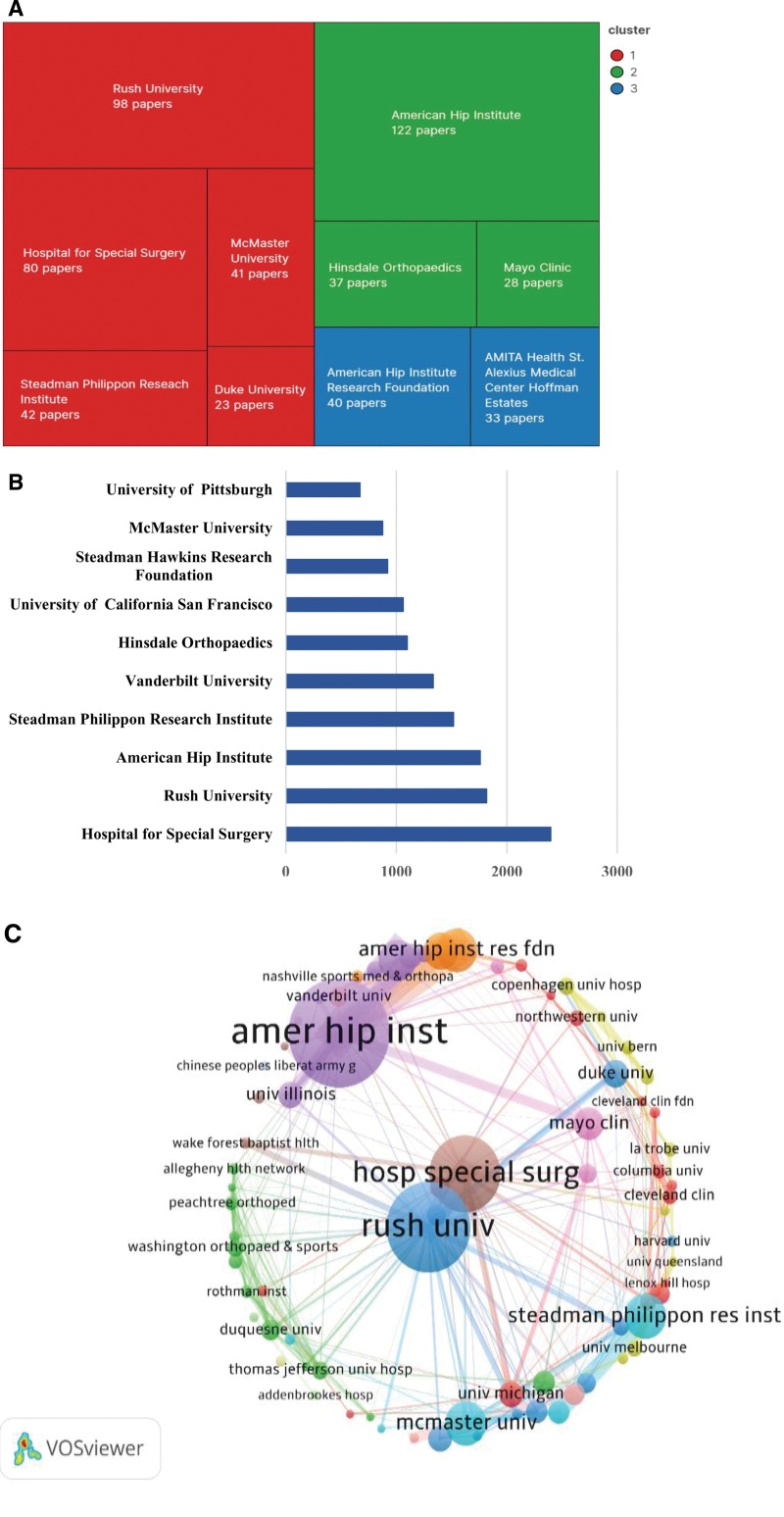
Highest impact institutions on arthroscopic hip. (A) The top 10 institutions in publications, the same color represent the same grouping. (B) The top 10 institutions in citations. (C) Network map of bibliometric co-authorship between institutions with 6 or more publications (n = 77). The thickness of the lines indicates the strength of the relationship, and the size of the node represents the number of published articles.

A total of 982 institutions have contributed to the identified literature. Top 10 institutions, predominantly from the United States, include: American Hip Institute (United States, n = 122), Rush University (United States, n = 98), Hospital for Special Surgery (United States, n = 80), Steadman Philippon Research Institute (United States, n = 42), American Hip Institute Research Foundation (United States, n = 40), Hinsdale Orthopedics (United States, n = 37), AMITA Health St. Alexius Medical Center Hoffman Estates (United States, n = 33), Mayo Clinic (United States, n = 28), Duke University (United States, n = 23) (Fig. 2A). There were 9 institutions from the United States Among the top 10 institutions. American institutions were the most active in hip arthroscopy research. The articles published by Hospital for Special Surgery have been cited the most (2399 times), followed by those from Rush University (1817 citations), and American Hip Institute (1760 citations) (Fig. 2B).

Using VOSviewer software, a visualized network of co-authorship relationships between institutions was created. This network displays only the 77 institutions with at least 6 articles. As the network reveals, the American Hip Institute has the strongest total link strength (n = 110), followed by Rush University (n = 65), Hospital for Special Surgery (n = 44), and American Hip Institute Research Foundation (n = 39). Rush University features the highest number of collaborators. Notably, the American Hip Institute collaborated closely with American Hip Institute Research Foundation and Hinsdale Orthopedics (Fig. 2C).

### 3.4. Journal of publication

In total, 147 journals published the identified 1094 articles. The top 20 journals account for 77.4% of the total publications (Table 1). The top 5 journals were *Arthroscopy-the Journal of Arthroscopic and Related Surgery, American Journal of Sports Medicine, Journal of Hip Preservation Surgery, Knee Surgery Sports Traumatology Arthroscopy, Arthroscopy Techniques.* Regarding total citation times, *Arthroscopy-the Journal of Arthroscopic and Related Surgery* ranked first with 8316 citations but *Hip: Aana Advanced Arthroscopic surgical Techniqures* was the last one. However, *Journal of Bone and Joint Surgery-British Volume* ranked first with 94.7 citations as for mean citation times. The top 20 journals average impact factor was 3.02, which indicates that a high reliability of the studies included the journals.

**Table 1:** Active journals on arthroscopy.

Journal	Articles	Total citations	Mean citations	Impact factor	Journal citation reports
Arthroscopy-The Journal of Arthroscopic and Related Surgery	262	8316	31.7	4.700	Q1
American Journal of Sports Medicine	132	3469	26.3	4.800	Q1
Journal of Hip Preservation Surgery	88	629	7.1	1.500	Q4
Knee Surgery Sports Traumatology Arthroscopy	47	790	16.8	3.800	Q2
Arthroscopy Techniques	43	174	4.0	NA	NA
Hip International	37	247	6.7	1.500	Q4
Clinical Orthopedics and Related Research	33	2001	60.6	4.300	Q2
Orthopedic Journal of Sports Medicine	33	114	3.5	2.600	Q3
Orthopedics	29	556	19.2	1.100	Q4
Journal of Bone and Joint Surgery-American Volume	22	1090	49.5	5.300	Q1
Clinics in Sports Medicine	20	775	38.8	2.00	Q3
Journal of Arthroplasty	15	811	54.1	3.500	Q2
Journal of Pediatric Orthopedics	14	507	36.2	1.700	Q3
Journal of Bone and Joint Surgery-British Volume	13	1231	94.7	NA	NA
Sports Medicine and Arthroscopy Review	11	128	11.6	1.900	Q4
Archives of Orthopedic and Trauma Surgery	10	124	12.4	2.300	Q3
BMC Musculoskeletal Disorders	10	163	16.3	2.300	Q3
Hip: Aana Advanced Arthroscopic Surgical Techniques	10	0	0.0	NA	NA
Journal of the American Academy of Orthopedic Surgeons	9	73	8.1	3.200	Q1
Operative Techniques in Sports Medicine	9	66	7.3	0.300	Q4

### 3.5. Keywords analysis and study hotspot

**Figure 3.**
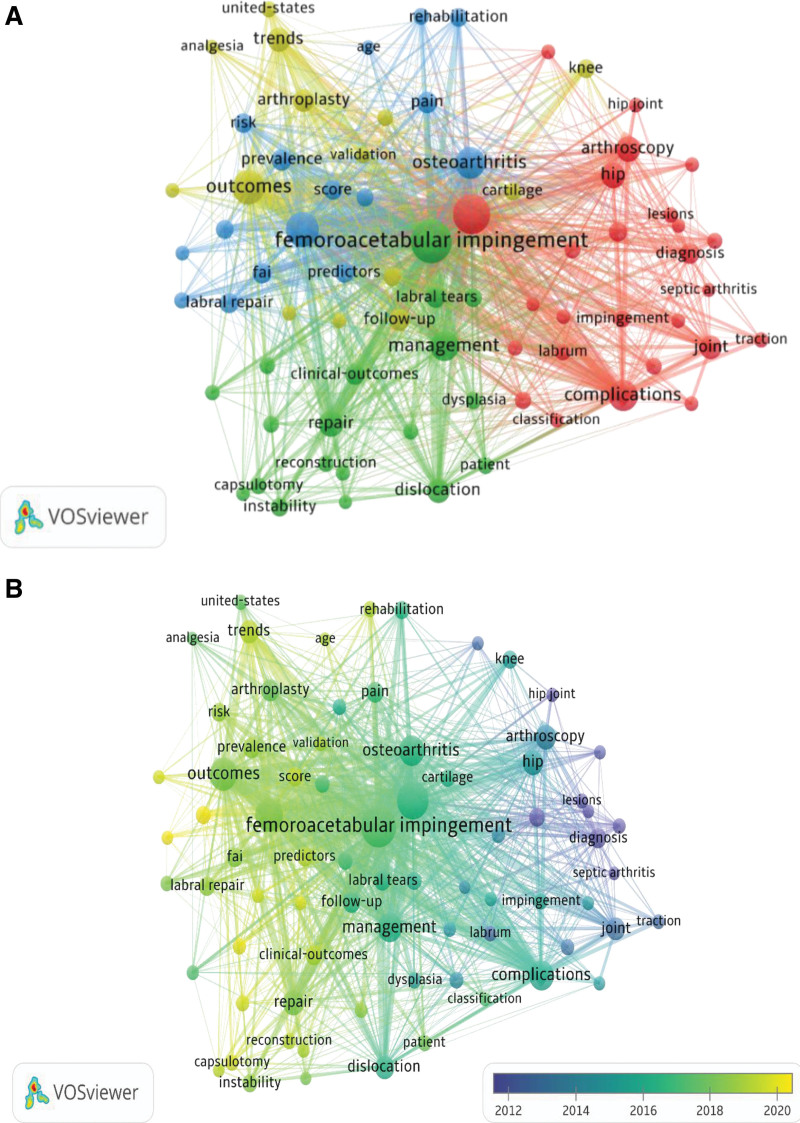
Co-occurrence analysis of keywords. (A) Keyword co-occurrence clustering network of hip arthroscopy. Each node in this map represents a keyword that occurred at least 20 times. Different colors indicated diverse research clusters, red for diagnosis cluster, green for basic research cluster and blue for clinical trial cluster. (B) Distribution of keywords according to average publication yr. Colors showed evolution of keyword over time (blue: earlier, yellow: later).

All keywords, inclusive of author keywords and keywords plus from articles on hip arthroscopy, which occurred at least 20 times were analyzed via the VOSviewer software (Fig. 3A). Out of the 1819 keywords, 75 met the threshold. They were segregated into 4 colored clusters: Cluster1 (red) encompassed 26 keywords related to diagnosis, such as “arthritis,” “impingement,” “hip arthroscopy,” “arthroscopy,” “lesions,” “abdominal compartment syndrome,” ”MR arthrography” or “surgical dislocation”; Cluster2 (green) consisted of 19 keywords, mainly concerning treatment, such as “capsular plication,” “capsulotomy,” “periacetabular osteotomy,” “reconstruction,” “repair,” “resection”; Cluster 3 (blue), including 16 keywords, related to preoperative evaluation, such as “predictors,” “age,” “prevalence,” “score,” “acceptable symptomatic state”; Cluster4 (yellow) involved 14 keywords, mainly focused on prognosis, such as “ survivorship,” “outcomes,” “trends,” “validation” or “follow-up.”

To understand the historical evolution of research hotspots, we assessed the co-occurrence of all keywords over time using VOSviewer and obtain a visual network diagram (Fig. 3B). The blue indicates that the keywords appeared early, on the contrary, the yellow represents a more current appearance. In the initial stages of research on hip arthroscopy, the main research hotspots were “septic arthritis,” “diagnosis,” “MR arthrography,” “acetabular labrum.” More recently, the keywords “femoroacetabular impingement syndrome,” “risk-factors,” “capsulotomy” and “score” have been gaining popularity in recently published articles.

**Figure 4.**
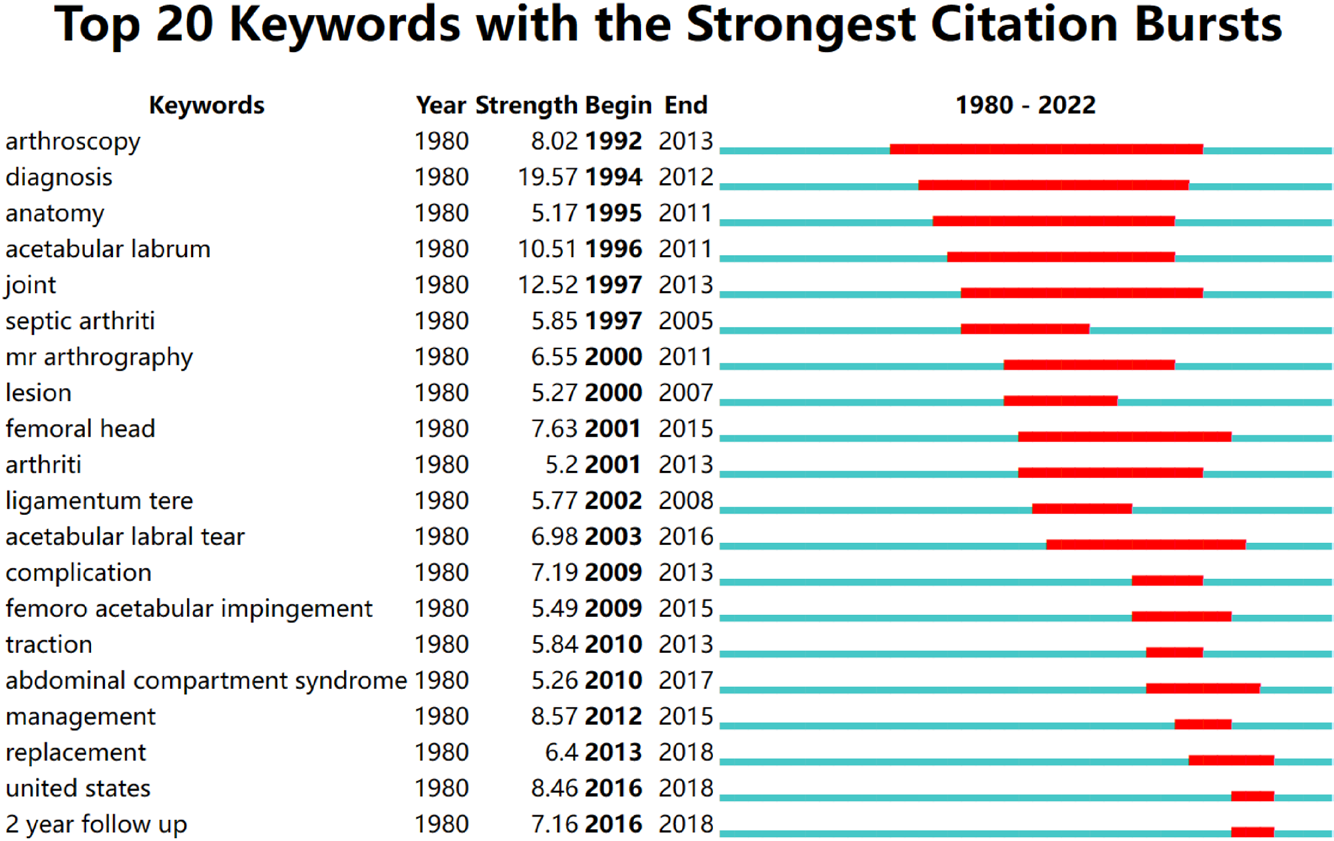
The top 20 keywords with the strongest citation bursts.

Figure 4 displays the top 20 keywords with the most potent citation bursts, the blue line signifies the base timeline, while the red line denotes the burst duration of a topic. The surge of keywords in this field commenced in 1999, with “diagnosis” (19.57) experiencing the strongest bursts, succeeded by “joint” (12.52) and “acetabular labrum” (10.51). Notably, “management,” “United States,” “arthroscopy,” “femoral head,” “complication” and “2-year follow-up” represent some of the current hotspots

**Figure 5.**
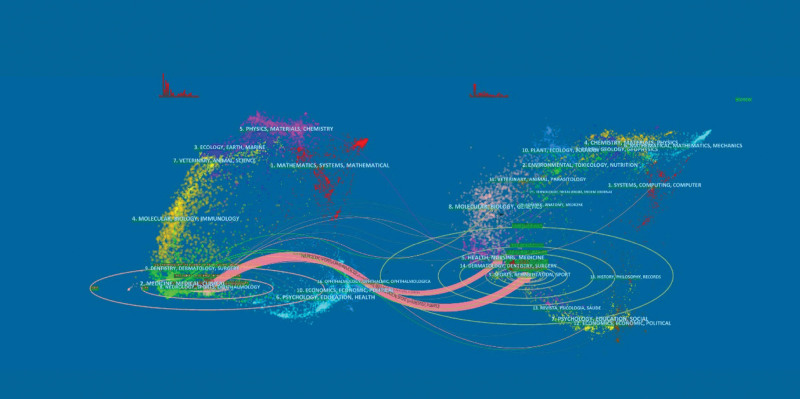
The dual-map overlay of journals pertaining to hip arthroscopy. Notes: On the left are the citing journals, while on the right are the cited journals, and the colored paths represent citation relationships.

As portrayed in Figure 5, the 2 orange paths are the primary citation paths, signifying that research published in neurology/sports/ophthalmology journals was principally cited by literature in health/nursing/medicine/sports/rehabilitation/sport journals.

**Figure 6.**
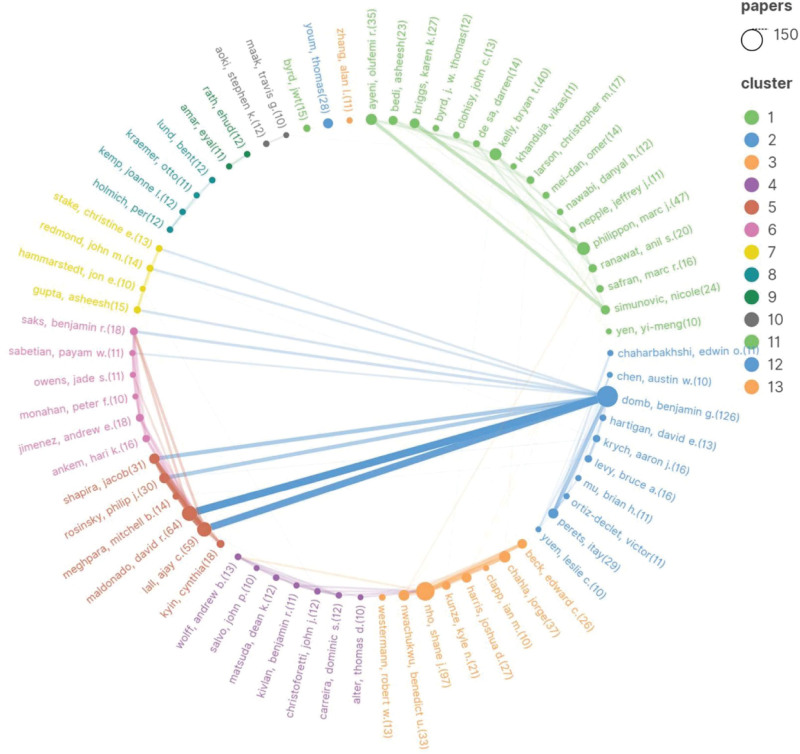
Author co-authorship network knowledge graph.

The authors’ articles on hip arthroscopy were analyzed using VOSviewer and further mapped with Scimago Graphica. Benjamin G Domb published the highest number of articles (126), followed by Shane J Nho, (97), David R Maldonado (64), and Ajay Lall (59). This collaboration network reveals that the authors generally worked together loosely, but there were also instances of close collaborations, such as Benjamin G Domb, David R Maldonado, and Ajay Lall (Fig. 6).

### 3.6. The top 100 cited articles

**Figure 7.**
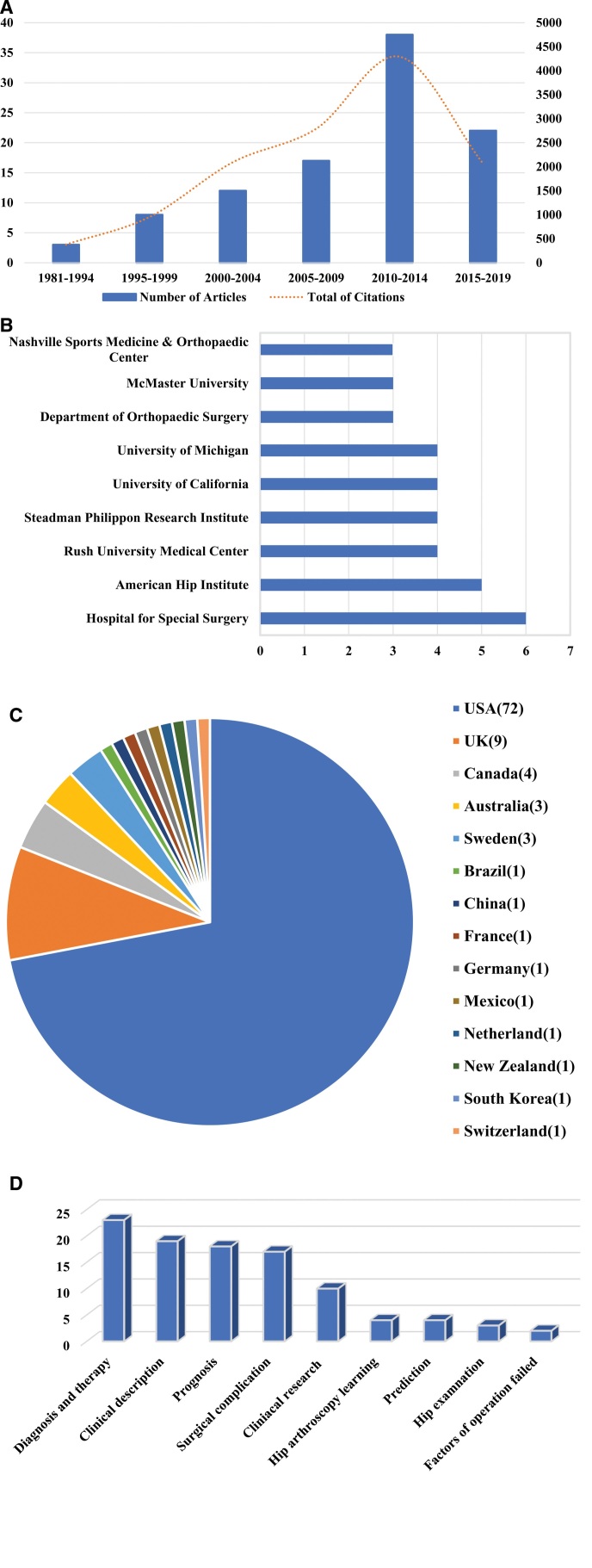
Top 100 most-cited publications on hip arthroscopy. (A) Yr of publications. (B) Institutions with > 2 articles. (C) Distribution of articles by original country. (D) Publication topics.

After ranking by the total number of citations, the top 100 most-cited articles were selected from 1094 articles published between 1981 and 2019 (Table 2). The period from 2010 to 2014 saw the highest number of articles published, with 38 articles, followed by the timespan from 2015 to 2019, which had 22 articles (Fig. 7A).

**Table 2:** The top 100 cited articles on hip arthroscopy.

Rank	Title	Author	Journal	Institution	Country	Yr	Total citation	Average per yr
1	Outcomes following hip arthroscopy for femoroacetabular impingement with associated chondrolabral dysfunction minimum 2-yr follow-up	Philippon MJ^[17]^	The Journal of Bone and Joint Surgery.British volume	Steadman Hawkins Research Foundation	USA	2009	597	42.64
2	Prospective analysis of hip arthroscopy with 2-yr follow-up	Byrd JW^[18]^	Arthroscopy: The Journal of Arthroscopic and Related Surgery	Vanderbilt University School of Medicine	USA	2000	393	17.09
3	Trends in Hip Arthroscopy Utilization in the United States	Bozic KJ^[19]^	The Journal of Arthroplasty	University of California	USA	2013	273	27.3
4	Revision hip arthroscopy	Philippon M J^[17]^	The American Journal of Sports Medicine	the Steadman Hawkins Research Foundation	USA	2007	269	16.81
5	Complications and reoperations during and after hip arthroscopy: a systematic review of 92 studies and more than 6000 patients	Harris JD	Arthroscopy: The Journal of Arthroscopic and Related Surgery	Rush University Medical Center	USA	2013	254	25.4
6	Trends and demographics in hip arthroscopy in the United States	Montgomery SR	Arthroscopy: The Journal of Arthroscopic and Related Surgery	University of California	USA	2013	244	24.4
7	Hip arthroscopy for acetabular labral tears	Farjo LA	Arthroscopy: The Journal of Arthroscopic and Related Surgery	University of California	USA	1999	241	10.04
8	Diagnostic accuracy of clinical assessment, magnetic-resonance-imaging, magnetic resonance arthrography, and intra-articular injection in hip arthroscopy patients	Byrd JW^[18]^	The American Journal of Sports Medicine	Nashville Sports Medicine & Orthopedic Center	USA	2004	240	12.63
9	Hip arthroscopy: Current indications, treatment options, and management issues	Kelly BT	The American Journal of Sports Medicine	University of Pittsburgh Medical Center	USA	2003	231	11.55
10	Hip arthroscopy: Complications in 1054 cases	Clarke MT	Clinical Orthopedics and Related Research	University of Cambridge	UK	2003	225	11.25
11	Radiologic and Intraoperative findings in revision hip arthroscopy	Heyworth BE	Arthroscopy: The Journal of Arthroscopic and Related Surgery	Hospital for Special Surgery	USA	2007	222	13.88
12	Hip arthroscopy utilizing the supine position	Byrd JW^[18]^	Arthroscopy: The Journal of Arthroscopic and Related Surgery	Southern Sports Medicine and Orthopedic Center	USA	1994	221	7.62
13	Hip arthroscopy vs best conservative care for the treatment of femoroacetabular impingement syndrome (UK FASHIoN): a multicentre randomized controlled trial	Griffin DR	Lancet	University of Warwick	UK	2018	219	43.8
14	Prospective Analysis of Hip Arthroscopy with 10-yr follow-up	Byrd JW^[18]^	Clinical Orthopedics and Related Research	Nashville Sports Medicine Foundation	USA	2010	214	16.46
15	Hip arthroscopy in the presence of dysplasia	Byrd JW^[18]^	Arthroscopy: The Journal of Arthroscopic and Related Surgery	Nashville Sports Medicine & Orthopedic Center	USA	2003	207	10.35
16	Evidence of validity for the hip outcome score in hip arthroscopy	Martin RL	Arthroscopy: The Journal of Arthroscopic and Related Surgery	Duquesne University	USA	2007	190	11.88
17	Hip arthroscopy for labral pathology: prospective analysis with 10-yr follow-up	Byrd JW^[18]^	Arthroscopy: The Journal of Arthroscopic and Related Surgery	Nashville Sports Medicine Foundation	USA	2009	186	13.29
18	Arthroscopy for labral tears in patients with developmental dysplasia of the hip: a cautionary note	Parvizi J	The Journal of Arthroplasty	Rothman Institute of Orthopedics at Thomas Jefferson University Hospital	USA	2009	181	12.93
19	Hip arthroscopy for femoroacetabular impingement in patients aged 50 yr or older	Philippon MJ^[17]^	Arthroscopy: The Journal of Arthroscopic and Related Surgery	Steadman Philippon Research Institute	USA	2012	170	15.45
20	Survivorship and outcomes 10 yr following hip arthroscopy for femoroacetabular impingement	Menge TJ	The Journal of Bone and Joint Surgery.American volume	Steadman Philippon Research Institute	USA	2017	164	27.33
21	Magnetic resonance arthrography vs arthroscopy in the evaluation of articular hip pathology	Keeney JA	Clinical Orthopedics and Related Research	Department of Orthopedic Surgery	USA	2004	162	8.53
22	Complications of hip arthroscopy	Sampson, TG	Clinics in Sports Medicine	St.Francis Memorial Hospital	USA	2001	159	7.23
23	Why do hip arthroscopy procedures fail?	Bogunovic L	Clinical Orthopedics and Related Research	Department of Orthopedic Surgery	USA	2013	157	15.7
24	Hip arthroscopy - an anatomic study of portal placement and relationship to the extraarticular structures	Byrd JW^[18]^	Arthroscopy: The Journal of Arthroscopic and Related Surgery	Southern Sports Medicine and Orthopedic Center	USA	1995	153	5.46
25	Catastrophic failure of hip arthroscopy due to iatrogenic instability: can partial division of the ligamentum teres and iliofemoral ligament cause subluxation?	Mei-Dan O	Arthroscopy: The Journal of Arthroscopic and Related Surgery	Millennium Institute of Sport and Health	New Zealand	2012	145	13.18
26	Anterior dislocation of the hip after arthroscopy in a patient with capsular laxity of the hip a case report	Ranawat AS	The Journal of Bone and Joint Surgery.American volume	Department of Orthopedic Surgery	USA	2009	140	10
27	The learning curve for hip arthroscopy: a systematic review	Hoppe DJ	Arthroscopy: The Journal of Arthroscopic and Related Surgery	Division of Orthopedic Surgery	USA	2014	136	15.11
28	Early outcomes after hip arthroscopy for femoroacetabular impingement in the athletic adolescent patient a preliminary report	Philippon MJ^[17]^	Journal of Pediatric Orthopedics	Steadman Hawkins Research Foundation	USA	2008	134	8.93
29	The role of hip arthroscopy in the diagnosis and treatment of hip disease	McCarthy JC	Orthopedics	New England Baptist Hospital	USA	1995	133	4.75
30	Hip arthroscopy in athletes 10-yr follow-up	Byrd JW^[18]^	The American Journal of Sports Medicine	The American Journal of Sports Medicine	USA	2009	133	9.5
31	What factors influence long-term survivorship after hip arthroscopy?	McCarthy JC	Clinical Orthopedics and Related Research	Harris Orthopedic Laboratory	USA	2011	133	11.08
32	Early outcome of hip arthroscopy for femoroacetabular impingement the role of femoral osteoplasty in symptomatic improvement	Bardakos NV	The Journal of Bone and Joint Surgery.British volume	The Richard Villar Practice The Wellington Hospital	UK	2008	129	8.6
33	Joint space predicts THA after hip arthroscopy in patients 50 yr and older	Philippon MJ^[17]^	Clinical Orthopedics and Related Research	Center for Outcomes-Based Orthopedic Research	USA	2013	129	12.9
34	Capsular management during hip arthroscopy: from femoroacetabular impingement to instability	Bedi A	Arthroscopy: The Journal of Arthroscopic and Related Surgery	University of Michigan	USA	2011	128	10.67
35	Tears of the ligamentum teres prevalence in hip arthroscopy using 2 classification systems	Botser IB	The American Journal of Sports Medicine	Hinsdale Orthopedic Associates	USA	2011	128	10.67
36	Trends in utilization and outcomes of hip arthroscopy in the United States between 2005 and 2013	Maradit Kremers H^[20]^	The Journal of Arthroplasty	Mayo Clinic	USA	2017	127	21.17
37	The relationship between diagnosis and outcome in arthroscopy of the hip	O’leary JA	Arthroscopy: The Journal of Arthroscopic and Related Surgery	Duke University Medical Center	USA	2001	123	5.59
38	Age-related trends in hip arthroscopy: a large cross-sectional analysis	Sing DC^[7]^	Arthroscopy: The Journal of Arthroscopic and Related Surgery	University of California-San Francisco	USA	2015	121	15.13
39	Patient-Reported Outcome questionnaires for hip arthroscopy: a systematic review of the psychometric evidence	Tijssen M	BMC Musculoskeletal Disorders	Sport Medisch Centrum Papendal	Netherland	2011	120	10
40	Hip arthroscopy outcomes with respect to patient acceptable symptomatic state and minimal clinically important difference	Levy DM	Arthroscopy: The Journal of Arthroscopic and Related Surgery	Rush University Medical Center	USA	2016	115	16.43
41	Does the modified Harris hip score reflect patient satisfaction after hip arthroscopy?	Aprato A	The American Journal of Sports Medicine	Spire Cambridge Lea Hospital	UK	2012	112	10.18
42	A geographic zone method to describe intra-articular pathology in hip arthroscopy: cadaveric study and preliminary report	Ilizaliturri VM Jr	Arthroscopy: The Journal of Arthroscopic and Related Surgery	National Rehabilitation Institute of Mexico	Mexico	2008	111	7.4
43	Outcomes 2–5 yr following hip arthroscopy for femoroacetabular impingement in the patient aged 11–16 yr	Philippon MJ^[17]^	Arthroscopy: The Journal of Arthroscopic and Related Surgery	Steadman Philippon Research Institute	USA	2012	111	10.09
44	Hip arthroscopy without traction: In vivo anatomy of the peripheral hip joint cavity	Dienst M	Arthroscopy: The Journal of Arthroscopic and Related Surgery	University Hospital	Germany	2001	110	5
45	The evidence for hip arthroscopy: grading the current indications	Stevens MS	Arthroscopy: The Journal of Arthroscopic and Related Surgery	Dalhousie University	Canada	2010	107	8.23
46	Hip arthroscopy: analysis of a single surgeon learning experience	Konan S	The Journal of Bone and Joint Surgery.American volume	University College London Hospital	UK	2011	103	8.58
47	Outcomes for hip arthroscopy according to sex and age a comparative matched-group analysis	Frank RM	The Journal of Bone and Joint Surgery.American volume	Rush University Medical Center	USA	2016	98	14
48	The safe zone for hip arthroscopy: a cadaveric assessment of central, peripheral, and lateral compartment portal placement	Robertson WJ	Arthroscopy: The Journal of Arthroscopic and Related Surgery	Hospital for Special Surgery	USA	2008	97	6.47
49	Systematic review and meta-analysis of outcomes after hip arthroscopy in femoroacetabular impingement	Minkara AA^[21]^	The American Journal of Sports Medicine	Columbia University Medical Center	USA	2019	97	24.25
50	Complications of arthroscopy of the hip	Griffin DR	The Journal of Bone and Joint Surgery.British volume	Addenbrooke NHS Trust	UK	1999	96	4
51	The outcome of hip arthroscopy in Australian football league players: a review of 27 hips	Singh PJ	Arthroscopy: The Journal of Arthroscopic and Related Surgery	Mercy and Belbird Private Hospitals	Australia	2010	96	7.38
52	Revision hip arthroscopy indications and outcomes: a systematic review	Sardana V	Arthroscopy: The Journal of Arthroscopic and Related Surgery	McMaster University	Canada	2015	96	12
53	The diagnostic accuracy of a clinical examination in determining intra-articular hip pain for potential hip arthroscopy candidates	Martin RL	Arthroscopy: The Journal of Arthroscopic and Related Surgery	Duquesne University	USA	2008	95	6.33
54	Predictors of hip arthroscopy outcomes for labral tears at minimum 2-yr follow-up: the influence of age and arthritis	McCormick F	Arthroscopy: The Journal of Arthroscopic and Related Surgery	Harvard Combined Orthopedic Residency Program	USA	2012	95	8.64
55	Clinical and radiographic predictors of intra-articular hip disease in arthroscopy	Nepple JJ	The American Journal of Sports Medicine	Washington University School of Medicine	USA	2011	94	7.83
56	Residual deformity is the most common reason for revision hip arthroscopy: a three-dimensional CT study	Ross JR^[3]^	Clinical Orthopedics and Related Research	University of Michigan	USA	2015	94	11.75
57	Diagnosis of the painful hip by magnetic-resonance-imaging and arthroscopy	Edwards DJ	The Journal of Bone and Joint Surgery.British volume	Addenbrooke Hospital	UK	1995	93	3.32
58	Diagnostic and operative arthroscopy of the hip	Eriksson E	Orthopedics	Karolinska University Hospital	Switzerland	1986	92	2.49
59	The incidence of heterotopic ossification after hip arthroscopy	Bedi A	The American Journal of Sports Medicine	University of Michigan	USA	2012	92	8.36
60	Hip arthroscopy in athletes	Byrd JW^[18]^	Clinics in Sports Medicine	Nashville Sports Medicine & Orthopedic Center	USA	2001	91	4.14
61	Hip arthroscopy surgical volume trends and 30-day postoperative complications	Cvetanovich GL	Arthroscopy: The Journal of Arthroscopic and Related Surgery	Rush University Medical Center	USA	2016	91	13
62	Complications in hip arthroscopy	Funke EL	Arthroscopy: The Journal of Arthroscopic and Related Surgery	Schulthess Hospital	Sweden	1996	89	3.3
63	Hip arthroscopy improves symptoms associated with FAI in selected adolescent athletes	Fabricant PD	Clinical Orthopedics and Related Research	Hospital for Special Surgery	USA	2012	89	8.09
64	Return to play after hip arthroscopy with microfracture in elite athletes	McDonald JE	Arthroscopy: The Journal of Arthroscopic and Related Surgery	Texas Orthopedics, Sports & Rehabilitation	USA	2013	87	8.7
65	Revision hip arthroscopy: a systematic review of diagnoses, operative findings, and outcomes	Cvetanovich GL	Arthroscopy: The Journal of Arthroscopic and Related Surgery	Rush University	USA	2015	87	10.88
66	Does primary hip arthroscopy result in improved clinical outcomes?: 2-yr clinical follow-up on a mixed group of 738 consecutive primary hip arthroscopies performed at a high-volume referral center	Gupta A	The American Journal of Sports Medicine	American Hip Institute	USA	2016	86	12.29
67	The demographic characteristics of high-level and recreational athletes undergoing hip arthroscopy for femoroacetabular impingement: a sports-specific analysis	Nawabi DH	Arthroscopy: The Journal of Arthroscopic and Related Surgery	Hospital for Special Surgery	USA	2014	83	9.22
68	Hip arthroscopy - the supine position	Byrd JW^[18]^	Clinics in Sports Medicine	Vanderbilt University School of Medicine	USA	2001	82	3.73
69	Labral tears, extraarticular injuries, and hip arthroscopy in the athlete	Bharam, S	Clinics in Sports Medicine	Lenox Hill Hospital	USA	2006	81	4.76
70	Combined hip arthroscopy and limited open osteochondroplasty for anterior femoroacetabular impingement	Clohisy JC	The Journal of Bone and Joint Surgery.American volume	Barnes-Jewish Hospital	USA	2010	81	6.23
71	An anatomical study of the acetabulum with clinical applications to hip arthroscopy	Philippon MJ^[17]^	The Journal of Bone and Joint Surgery.American volume	Steadman Philippon Research Institute	USA	2014	80	8.89
72	Hip arthroscopy in children and adolescents	Kocher MS	Journal of Pediatric Orthopedics	Harvard Medical School	USA	2005	79	4.39
73	Do complications in hip arthroscopy change with experience?	Souza BG	Arthroscopy: The Journal of Arthroscopic and Related Surgery	Medical Sciences Faculty	Brazil	2010	79	6.08
74	Hip arthroscopy for intra-articular pathology: a systematic review of outcomes with and without femoral osteoplasty	Kemp JL	British Journal of Sports Medicine	The University of Melbourne	Australia	2012	77	7
75	Hip chondropathy at arthroscopy: prevalence and relationship to labral pathology, femoroacetabular impingement and patient-reported outcomes	Kemp JL	British Journal of Sports Medicine	University of Queensland	Australia	2014	77	8.56
76	Complications and survival analyses of hip arthroscopies performed in the national health service in england: a review of 6395 cases	Malviya A	Arthroscopy: The Journal of Arthroscopic and Related Surgery	Northumbria Healthcare NHS Foundation Trust	UK	2015	77	9.63
77	How much arthritis is too much for hip arthroscopy: a systematic review	Domb BG	Arthroscopy: The Journal of Arthroscopic and Related Surgery	American Hip Institute	USA	2015	77	9.63
78	Evaluating hip labral tears using magnetic resonance arthrography: a prospective study comparing hip arthroscopy and magnetic resonance arthrography diagnosis	Chan YS	Arthroscopy: The Journal of Arthroscopic and Related Surgery	Chang-Gung Memorial Hospital	China	2005	76	4.22
79	Arthroscopy of the hip: 12 yr of experience	Dorfmann H	Arthroscopy: The Journal of Arthroscopic and Related Surgery	Center Hospitalier Robert Ballanger	France	1999	75	3.13
80	Radiographic comparison of surgical hip dislocation and hip arthroscopy for treatment of cam deformity in femoroacetabular impingement	Bedi A	The American Journal of Sports Medicine	University of Michigan	USA	2011	73	6.08
81	Is diagnostic arthroscopy of the hip worthwhile? A prospective review of 328 adults investigated for hip pain	Baber YF	The Journal of Bone and Joint Surgery.British volume	Addenbrooke Hospital	UK	1999	72	3
82	Hip arthroscopy for labral tears review of clinical outcomes with 4.8-yr mean follow-up	Kamath AF	The American Journal of Sports Medicine	Hospital of University of Pennsylvania	USA	2009	72	5.14
83	Total dislocation of the hip joint after arthroscopy and ileopsoas tenotomy	Sansone M	Knee Surgery Sports Traumatology Arthroscoopy	Sahlgrenska University Hospital	Sweden	2013	72	7.2
84	The role of hip arthroscopy in the elite athlete	McCarthy J	Clinical Orthopedics and Related Research	Clinical Orthopedics and Related Research	USA	2003	71	3.55
85	Hip dislocation or subluxation after hip arthroscopy: a systematic review	Duplantier NL	Arthroscopy: The Journal of Arthroscopic and Related Surgery	Houston Methodist Orthopedic & Sports Medicine	USA	2016	68	9.71
86	Arthroscopy of the hip in juvenile chronic arthritis	Holgersson S	Journal of Pediatric Orthopedics	Lund University Hospital	Sweden	1981	67	1.6
87	Safety measures in hip arthroscopy and their efficacy in minimizing complications: a systematic review of the evidence	Gupta A	Arthroscopy: The Journal of Arthroscopic and Related Surgery	American Hip Institute	USA	2014	67	7.44
88	Evidence of capsular defect following hip arthroscopy	McCormick F	Knee Surgery Sports Traumatology Arthroscoopy	Rush University	USA	2014	67	7.44
89	Defining the learning curve for hip arthroscopy a threshold analysis of the volume-outcomes relationship	Mehta N	The American Journal of Sports Medicine	Hospital for Special Surgery	USA	2018	66	13.2
90	Labral penetration rate in a consecutive series of 300 hip arthroscopies	Domb B	The American Journal of Sports Medicine	Hinsdale Orthopedics Associates	USA	2012	65	5.91
91	Learning curve of basic hip arthroscopy technique: CUSUM analysis	Lee YK	Knee Surgery Sports Traumatology Arthroscoopy	Seoul National University Bundang Hospital	South Korea	2013	65	6.5
92	Use of hip arthroscopy and risk of conversion to total hip arthroplasty: a population-based analysis	Schairer WW	Arthroscopy: The Journal of Arthroscopic and Related Surgery	Hospital for Special Surgery	USA	2016	65	9.29
93	Outcomes of hip arthroscopy in the older adult: a systematic review of the literature	Griffin DW	The American Journal of Sports Medicine	Walter Reed National Military Medical Center	USA	2017	65	10.83
94	Hip arthroscopy in patients age 40 or older: a systematic review	Horner NS	Arthroscopy: The Journal of Arthroscopic and Related Surgery	McMaster University	Canada	2017	64	10.67
95	Predictors of clinical outcomes after hip arthroscopy a prospective analysis of 1038 patients with 2-yr follow-up	Domb BG	The American Journal of Sports Medicine	American Hip Institute	USA	2018	64	12.8
96	Patient-reported outcomes of capsular repair vs capsulotomy in patients undergoing hip arthroscopy: minimum 5-yr follow-up-a matched comparison study	Domb BG	Arthroscopy: The Journal of Arthroscopic and Related Surgery	American Hip Institute	USA	2018	64	12.8
97	Rehabilitation after hip femoroacetabular impingement arthroscopy	Wahoff M	Clinics in Sports Medicine	Howard Head Sports Medicine	USA	2011	63	5.25
98	Complications following hip arthroscopy: a systematic review and meta-analysis	Kowalczuk M	Knee Surgery Sports Traumatology Arthroscoopy	McMaster University	Canada	2013	63	6.3
99	Patient and disease characteristics associated with hip arthroscopy failure in acetabular dysplasia	Ross JR^[3]^	The Journal of Arthroplasty	Broward Orthopedic Specialists	USA	2014	63	7
100	Complications in hip arthroscopy: a systematic review and strategies for prevention	Weber AE	Sports Medicine and Arthroscopy Review	University of Michigan Hospital	USA	2015	63	7.88

In terms of the top 100 most-cited articles, the Hospital for Special Surgery contributed the most articles related hip arthroscopic research, with 6 articles, followed by American Hip Institute, featuring 5 articles (Fig. 7B).

The majority of the top 100 cited articles originated from United States, United Kingdom, Canada, Australia and Sweden, and account for 91% of all articles. They were followed by Brazil, China, France, Germany, Mexico, Netherlands, New Zealand, South Korea and Switzerland with 1 article each (Fig. 7C). Notably, only China and South Korea represented Asian in contributing to these top 100 cited articles.

The top 5 scientific journals published the majority of the top 100 cited articles, including *Arthroscopy-The Journal of Arthroscopic and Related Surgery* with 41 articles, *The American Journal of Sports Medicine* with 16 articles, *Clinical Orthopedics and Related Research* with 9 articles, *Clinics in Sports Medicine* with 5 articles, and *Knee Surgery Sports Traumatology Arthroscopy* with 4 articles (Table 3). Although the number of articles published by *The American Journal of Sports Medicine* ranked second in the number of articles published, it held the first rank in impact factor, indicating a more extensive influence compared to other journal on the list.

**Table 3:** Journal with at least 1 of the most-cited articles on hip arthroscopy.

Journal	Articles	Total citations	Mean citations	Impact factor
Arthroscopy-The Journal of Arthroscopic and Related Surgery	41	5316	129.7	4.772
The American Journal of Sports Medicine	16	1887	117.9	6.202
Clinical Orthopedics and Related Research	9	1274	141.6	4.176
Clinics in Sports Medicine	5	476	95.2	2.182
Knee Surgery Sports Traumatology Arthroscopy	4	267	66.8	4.342

The most common research focus on diagnosis and therapy (23 articles), followed by clinical description (19 articles), Prognosis (18 articles), Surgical complication (17 articles), Clinical Research (10 articles), Hip arthroscopy learning (4 articles), Prediction (4 articles), Hip examination (3 articles), Factors of operation failed (2 articles) (Fig. 7D).

## 4. Discussion

Hip arthroscopy has gained increasing popularity over the last 20 years.^[19,22]^ It has been successfully employed in the diagnosis and treatment of various hip pathologies.^[23–26]^ The use of hip arthroscopy surged 5-fold from 2005 to 2013, making it one of the fasting-growing fields of arthroscopic surgery.^[20]^ Some advantages of hip arthroscopy include lower rates of complications and reoperations,^[4,21]^ which have led to a high rate of return to sport and work.^[27,28]^ Considering the numerous advantages and rapid development of hip arthroscopy, this study aims to conduct a bibliometric analysis of hip arthroscopy, exploring and identifying the research foci and trends, as well as establishing a knowledge base for hip arthroscopy. It is hoped that this study will enlighten scientific researchers about the current research scenario in hip arthroscopy and provide future research directions.

### 4.1. Publication trends in hip arthroscopy scientific literature

The number of articles related to hip arthroscopy has seen a swift rise over the last 10 years, suggesting heightened interest among researchers. In terms of geographic distribution, the United States leads in publishing the highest number of articles on hip arthroscopic research, reflecting its leadership status in this area. While America spearheads the total number of articles on hip arthroscopy, Europe, Asia, Oceania and Africa have also made tremendous contributions. This illustrates that hip arthroscopy is a globally significant topic attracting international research interest.

In terms of institutional contributions, the American Hip Institute from the United States ranks first among all institutions for the number of articles published. The Hospital for Special Surgery, also located in the United States, has accumulated the highest total citations. This reality helps explain why the United States is a pioneer in this field. These institutions undoubtedly play an important role in hip arthroscopy research. The collaborative network among countries and institutions shows an encouraging trend toward greater communication between regions. The United States, leading both economically and scientifically, has fostered close collaborations with Israel and Canada. As possess adequate funding, advanced equipment, and professional researchers, these developed countries and institutions are more likely to carry out studies related to hip arthroscopy. Researchers interested in hip arthroscopy may consider partnering with these organization.

The top 5 prolific journals on hip arthroscopy include *Arthroscopy-the Journal of Arthroscopic and Related Surgery, American Journal of Sports Medicine, Journal of Hip Preservation Surgery, Knee Surgery Sports Traumatology Arthroscopy and Arthroscopy Techniques*- 3 of which originate from the USA. This reflects that the United States places significant emphasis on healthcare and boasts superior scientific research capabilities. In addition, 3 of these journals have an impact factor (IF) >4. Therefore, when submitting articles on hip arthroscopy research, authors should give these journals more consideration.

### 4.2. Research focuses

The analysis of keywords in the field reveals that terms such as hip arthroscopy, femoroacetabular impingement, hip arthroscopy, surgery, and osteoarthritis have emerged and continue to be central themes. Prior to 2012, keywords included MR arthrography, traction, hip joint, diagnosis, indicating a primary focus on diagnosis and treatment. In more recent years, frequently used keywords such as acceptable symptomatic state, patient-reported outcomes, femoroacetabular impingement, revision, and highlight increased attention to prognosis.

### 4.3. The most influential publications

The most-cited article in hip arthroscopy, boasting a total citation count of 579 and an annual citation rate of 42.64, was published in *The Journal of Bone and Joint Surgery* (British volume) in 2009. Authored by Philippon MJ et al, “Outcomes following hip arthroscopy for femoroacetabular impingement with associated chondrolabral dysfunction: minimum 2-year follow-up,” evaluates the efficacy of hip arthroscopy for femoroacetabular impingement.^[17]^ In this research, they concluded that patients very highly satisfied with the arthroscopic surgery of the hip for FAI, achieved excellent hip function.

The second most-cited article, titled “Prospective Analysis of Hip Arthroscopy With 2-Year Follow-up” was published in *the Arthroscopy: The Journal of Arthroscopic and Related Surgery* by Byrd JW and Jones KS in 2000.^[18]^ To shift researchers’ attention from indications to outcomes of hip arthroscopy, the authors collected data from 38 cases of hip arthroscopic surgery and examined postoperative situations. They concluded that while complications from arthroscopic surgery were not insignificant, they were acceptable for appropriate patients. Their studies acted as an impetus for further investigative research into hip arthroscopic surgery.

The third most-cited article in the top-100 list is titled “Trends in hip arthroscopy utilization in the United States,” published in *The Journal of Arthroplasty* by Bozic KJ et al in 2013.^[19]^ In this study, the authors used the American Board of Orthopedic Surgery (ABOS) database to retrospectively analyze the annual occurrence rate of hip arthroscopic surgery from 2006 to 2010. They also calculated and compared the complication rate of hip arthroscopic surgeries performed by newly trained surgeons taking the ABOS Part II Board exam with complication rates reported in published articles. The results showed comparatively low reported complication rates. Moreover, the incidence of hip arthroscopic surgery displayed a sharp increase, rising from 83 in 2006 to 636 in 2010.

### 4.4. Limitations

There are several limitations to our study that should be confessed. First, our data related to hip arthroscopy was solely obtained from the Web of Science Core Collection database, excluding other databases such as PubMed, Scopus and Google Scholar. Consequently, some key articles may have been overlooked, potentially resulting in an incomplete or superficial understanding. Second, we only selected publications between 1900 and 2022. Given that the database will theoretically evolve in the future, our analysis results may slightly deviate from the actual landscape. Third, non-English language articles were excluded, which may mean neglecting some valuable information.

## 5. Conclusion

This study demonstrates a fast increase in the number of published articles associated with hip arthroscopy over the last decade. The United States has emerged as a leader among other nations in terms of the number of scientific articles published. The American Hip Institute, Rush University and Hospital for Special Surgery have been the most productive organizations conducting research related to hip arthroscopy. The top 5 prolific journals publishing on the topic include *Arthroscopy-the Journal of Arthroscopic and related Surgery, American Journal of Sports Medicine, Journal of Hip Preservation Surgery, Knee Surgery Sports Traumatology Arthroscopy, and Arthroscopy Techniques*. Research hotspots receiving the most attention have been diagnosis and therapy, clinical description, and prognosis. Researchers interested in hip arthroscopy are encouraged to familiarize themselves with not only the top 100 articles but also the broader scope of this comprehensive analysis. It integral to have a deep understanding of these influential works and the trends they represent in this rapidly growing field.

## Author contributions

**Conceptualization:** Lichen Ji, Senbo Zhu.

**Data curation:** Jinlong Tian, Yanlei Li, Xugang Zhong.

**Formal analysis:** Yanlei Li, Lichen Ji.

**Investigation:** Jinlong Tian, Yanlei Li, Lichen Ji.

**Methodology:** Yu Tong, Senbo Zhu, Yao Kang.

**Project administration:** Yao Kang.

**Software:** Yu Tong.

**Supervision:** Yu Tong, Wei Zhang, Xugang Zhong, Senbo Zhu, Yao Kang, Qing Bi.

**Validation:** Jinlong Tian, Qing Bi.

**Visualization:** Wei Zhang, Xugang Zhong, Qing Bi.

**Writing – original draft:** Jinlong Tian, Wei Zhang, Senbo Zhu.
